# Full-duplex multi-user MIMO communication systems performance optimization using leakage-based precoding

**DOI:** 10.1038/s41598-023-35409-9

**Published:** 2023-05-23

**Authors:** Merhawit Berhane Teklu, Dong-You Choi, Wei-Xiao Meng

**Affiliations:** 1 Department of Information and Communication Engineering, Chosun University, Gwangju, 61452 South Korea; 2 School of Electronics and Information Engineering, Harbin Institute of Technology, Harbin, 150080 China

**Keywords:** Energy science and technology, Engineering

## Abstract

The spectral efficiency (SE) can approximately double when using full-duplex (FD) multiuser MIMO communications. However, there are difficulties because of multiuser interferences, self-interference (SI), and co-channel interference (CCI). To improve the SE of the downlink (DL), this paper proposes CCI-aware enhancement to SLNR (signal-to-leakage-and-noise-ratio) signal-to-leakage-and-noise-ratio (SLNR). It considers a suppressing filter at the receiver to cancel the interferences again designing a beamformer based on CCI-plus-noise covariance matrices for every user at the transmitting side. Additionally, we propose an improvement in the SLNR method by using SI-plus-noise covariance matrices to design uplink (UL) beamformers. Unlike zero-forcing and block-diagonalization, the SLNR approach serves numerous antennas at users and BS (base station). The total SE of the communication yielded using the optimized precoder, i.e., obtained from the SLNR-based precoding. To achieve maximum energy efficiency (EE), we use a power consumption model. Simulation results confirm that full-duplex performs well compared to half-duplex (HD) when the number of antennas at every user in uplink as well downlink channels grow, for all Rician factors, for slight powers of the CCI and SI, and a limited number of antennas at the BS. With the proposed scheme for given transmit power and circuit power, we demonstrate that FD has a higher EE than HD.

## Introduction

Recently, it is widely recognized that multi-user (MU) multi-input-multi-output (MIMO) systems have the ability in improving wireless systems capacity^1,2^. Currently, multi-user MIMO wireless communication systems convey in a half-duplex way of communication this indicates uplink and downlink channel users operate in both directions by separating the time slots or frequencies, namely, frequency division duplexing (FDD) or time division duplexing (TDD), respectively. To exploit the current resources, it is necessary to use efficient communication techniques. Full duplex (FD) cellular system is becoming the key promising method for the future trends of mobile communications. This is because it has the potential to approximately double the spectral efficiency compared with the traditional half-duplex (HD) mode of communication^3–5^. However, in the FD-based system, there is a self-interference (SI) from the transmitter to the receiver at the base station (BS) and it is a pivotal obstruction that degrades the SE of the communication system. Hence, several techniques are being proposed to substantially suppress the SI and then use the FD mode of communication for different applications. Some of the SI mitigation techniques are antenna cancellation combined with RF interference cancellation^6^, analog cancellation^7^ and digital baseband interference suppression^8^. Insightful current studies about the SI cancellation in FD devices illustrated that SI is able remarkably mitigated to make the residual SI power as low as the noise floor^9–12^. This positive result shows that FD-based systems can be employed in practical scenarios.

### Related works

Several precoding methods were studied for the suppression of the SI for FD single-user MIMO systems (see^13–15^). Moreover, FD multi-user MIMO communication where uplink users transmit signals to the BS and downlink users receive signals from the base station at the same frequencies and times are analyzed in^16–22^. Subsequently, the FD-MU-MIMO system takes into account co-channel interference (CCI) from UL to DL, SI from DL to UL, and multi-user interference (MUI) in both channels sum-rates was studied in^23–25^. The authors in^24^ use ZF-BF (zero-forcing beamformer) and ZF-R (ZF receiver) for downlink and uplink communications, sequentially to maximize the sum rates of both channels. On the other hand, the authors in^25^ utilize a block diagonalization (BD) precoder and a block diagonalization receiving filter for downlink transmission and uplink reception, respectively. The researchers in^18,22,23^ considered users of both channels employed a single antenna. Hence, it is necessary to study Full-duplex MU-MIMO communication systems in which users at both channels are equipped with numerous antennas. Moreover, the BD and ZF beamforming schemes in^16–19,22–25^ are low complexes sub-optimal transmission techniques and cancel the MUI completely. Although these schemes are low complex to implement, for several applications of multi-user MIMO systems, there are constraints on the employment antennas at users as well as the base station. Furthermore, these schemes ignore the background noise component and suffer from noise enhancement, especially in the lower SNR range. Hence, SINR precoding was introduced in^26,27^ which makes a balance between eliminating the MUI and the noise to achieve the best system performance. Nevertheless, the solution can only be obtained iteratively because of the complexity and coupled nature of the optimization problem. Thus, signal-to-leakages-ratio (SLR) techniques were investigated for downlink multiuser MIMO communications to overcome the limitations of these precoding approaches in^28–30^. Consequently, this scheme realizes a better trade-off between performance and complexity. This makes a balance between the cancellation of the MUI and noise. Furthermore, this method maximizes the desired power for every user meantime, minimizes the power of the leakage added by this user for all other users. The coefficients of the precoder for all users are concurrently maximized using the SLNR method. This metric can decouple the optimization problems again and provide closed-form solutions. The employment of an SLNR-precoding scheme for a Full-duplex mode of communication is an encouraging approach for upcoming generation wireless systems^31–33^. This is because it substantially increases the SE without any limitation on the number of antenna configurations at the BS and users.

In this study, for the DL transmission, a CCI-aware advancement to the SLNR beamforming approach is proposed. This approach applies a Principal Component Analysis (PCA) suppression filter at the receiver to cancel the interferences as well as design a precoder using CCI-plus-noise covariance matrices for every user at the base station. In addition, this study proposes self-interference-aware advancement based on the SLNR precoding method for the uplink system and designs a precoder based on the SI-plus-noise covariance matrix. Further, this system utilizes a suppressing filter at the receiver to mitigate the interferences. In^28–30^, the SLNR-based precoding scheme is only implemented for DL MU-MIMO systems, however, this paper proposes for Full-duplex multiuser MIMO way of a communication system.

Besides, the SE of Full-duplex multiuser MIMO communication for independent and identical distributed (i.i.d) Rayleigh fading environment or Gaussian distribution has widely analyzed in^14–19,22–25^. Additionally, in recent studies^12,22^, the authors consider Rayleigh fading model, which is suitable for modeling rich scattering environments. But, in day-to-day activities, there exists a line-of-sight (LOS) path between the receiver as well transmitter, e.g., in mmWave and short-range systems. This implies that Rayleigh fading fails to capture the presence of LOS the dominant feature of future wireless systems operating by the candidate millimeter-wave (mmWave) systems. For the sake of overcoming such scenarios, this paper explores the Rician fading environment, i.e., formulating the channel which constitutes LOS plus non-line-of-sight (NLOS) elements.

Energy Efficiency (EE) is another crucial performance metric to consider while developing wireless communication systems for 5G and beyond, in addition to spectral efficiency. It is caused by an increasing disparity between battery capacity and signal processing circuit power consumption. Because FD systems simultaneously receive and transmit signals, they require more energy to cancel self-interference and co-channel interference^34,35^. As a result, the EE of a Full-duplex can be exceeded by the Half-duplex system if the precoder/beamformer is not designed correctly. In most related studies, the SE was maximized using ZF/BD precoders^16–21^ or SLNR-based schemes^31,32^. This work is an extension of^32^ and^33^, EE maximization was not explored, only SE was optimized for the system model that considered MUI and SI interferences as shown in^32^ as well as the FD-based system in^33^ which took into account the MUI, CCI, and SI interferences. Motivated by the above-related works, our study further investigates the FD-MU-MIMO system’s performance as stated in the following subsection.

### Main contributions

Our research investigates SLNR-based precoding techniques to greatly enhance the spectral efficiency and energy efficiency of Full-duplex multi-user MIMO comparison with Half-duplex multi-user MIMO communications. The main contributions are summarized as described as:

- The investigation of sum rates is derived by taking into account CCI for the downlink channel, SI for the uplink channel, and MUI at both channels for multiple streams per user in the flat Rician fading channel.
- For the DL communication, co-channel interference aware improvement to the SLNR scheme is developed. This system utilizes a PCA (Principal Component Analysis) whitening filter at the receiver for interference suppression and designing a precoder by making use of the CCI-plus-noise covariance matrix.
- For the uplink channel, an SI-aware enhancement to the SLNR precoding technique and designing a new precoder using the self-interference-plus-noise covariance matrix is proposed. Additionally, this system uses a mitigation filter for interference suppression.
- The spectral efficiencies for both downlink and uplink channels are obtained using the optimized precoders that are found in the proposed SLNR-based precoding schemes. Then, a new total SE of the Full-duplex multi-user MIMO mode of transmission is attained by adding the sum rates of both channels.
- Based on appropriate precoder design and circuit power consumption, the overall EE is maximized.
- The simulation results demonstrate that the SE of full-duplex regard to SLNR-precoding technique performs well compared to an HD system when the number of antennas at every user grows in UL and DL channels, for a small transmitted power at UL users and the BS, and all Rician factors. We also demonstrated that the achievable EE of FD is higher than the existing HD system when employing the proposed Precoding strategy for the effects of transmit power and circuit power consumption.

The remainder of the paper is organized as follows. The detailed system model descriptions for downlink and uplink channels are provided in section “System models”. Section “Proposed SLNR precoding scheme and problem formulations” presents the proposed optimization problem formulations to design the precoder coefficients for UL and DL channels by relying on the SLNR metric. Then, the total spectral efficiency of the Full-duplex Multiuser MIMO communications is attained by equating the optimized precoder to the sum-rate equations of UL and DL channels. Section “Energy efficiency maximization” presents the power consumption model, Problem Formulation, and Maximization of EE. Section “Simulation results and analysis” describes simulation results working on Matlab to evaluate the SE of Half-duplex and Full-duplex communications. Finally, section “Conclusion” presents concluding remarks.

*Notations*: Bold lowercase and uppercase symbols or letters represent vectors and matrices, respectively. ${\mathbf {I}_{N}}$ stands for the ${N\times {N}}$ identity matrix. ${(.)^{-1}}$ and ${(.)^{H}}$ designate matrix inversion and hermitian, sequentially. |.| denotes determinant operator. ${\mathbb {E}[.]}$ and ${\mathrm {Tr}(.)}$ are mathematical expectation operators and trace, respectively.

## System models

**Figure 1:**
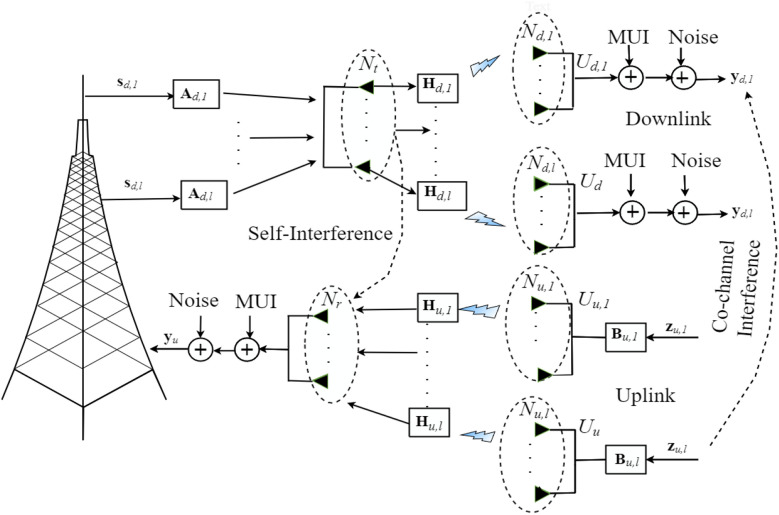
A system model for single cell Full-duplex Multi-user MIMO communications.

As shown in Fig. 1, we consider Full-duplex based base-station. From the figure, the BS employs a total of $N_{TR}=N_t+N_r$ antennas, where $N_t$ represents the number of transmitting antennas to transmit multiple data streams to DL $U_{d}$ users and $N_r$ denotes the number of receiving antennas to receive multiple data streams from UL $U_{u}$ users. The number of antennas at every user in DL and UL channels are represented by $N_{d,l}, l=1,2,\ldots \,,U_{d}$ and $N_{u,l}, l=1,2,\ldots \,,U_{u}$, respectively. We assumed Rician flat fading channel and this comprised of specular and scattered components^36–38^. The channel components can be described by $\mathbf {H}_{r,l}=\sqrt{\frac{{K}}{K+1}}{\bar{\mathbf {H}}_{r,l}}+\sqrt{\frac{{1}}{K+1}}{\tilde{\mathbf {H}}_{r,l}}, ~l=1,2,\ldots \,,U_{r}$. The representation of *r* depends on the downlink and uplink channels. *K* stands for the Rician factor, $\bar{\mathbf {H}}_{r,l}$ denotes the deterministic matrices with all of the entries magnitude of ones. As well, $\tilde{\mathbf {H}}_{r,l}$ is the Rayleigh component with entries i.i.d complex Gaussian random variables with zero-mean unit-variance, i.e., $\mathscr{CN}({0},{\mathbf {I}_{{N}_{r,l}}})$. Further, we assume that perfect channel state information at channel users and BS. The detailed mathematical model formulations for the FD-based MU-MIMO scenario are provided in the next subsections.

### Downlink signal model

By taking into account the aforementioned design considerations, the received vector at the $l\mathrm{th}$ user is defined by

1 $$\begin{aligned} \mathbf {y}_{d,l}=\mathbf {H}_{d,l}\mathbf {x}+\mathbf {H}_{cci,l}\mathbf {x}_{cci,l}+\mathbf {v}_{d,l} \end{aligned}$$

where $\mathbf {y}_{d,l}\in \mathscr {C}^{{N}_{d,l}\times {1}}$ is the signal received by user *l*; $\mathbf {H}_{d,l}\in \mathscr {C}^{{N}_{d,l}\times {{N}_{t}}}$ represents the channel matrix; $\mathbf {x}\in \mathscr {C}^{{N}_{t}\times {1}}$ denotes the transmitted signal vector; $\mathbf {H}_{cci,l}\in \mathscr {C}^{{N}_{d,l}\times {{N}_{u,l}}}$ and $\mathbf {x}_{cci,l}\in \mathscr {C}^{{N}_{u,l}\times {1}}$ are the channel matrix and transmitted signal vector of the co-channel interference, respectively; and $\mathbf {v}_{d,l}\in \mathscr {C}^{{N}_{d,l}\times {1}}$ denotes the noise vector distributed as $\mathscr{CN}\mathscr{}({0},{\sigma _{v}^2\mathbf {I}_{{N}_{d,l}}})$. The data streams and noise are assumed to be statistically independent.

We denote the data streams for the $l\mathrm{th}$ user by a vector $\mathbf {s}_{d,l}$ and this can be multiplied by a precoding matrix $\mathbf {A}_{d,l}\in \mathscr {C}^{{N}_{t}\times {n}}$, where *n* represents the number of streams at every user and we assume that $n\le {N}_{d,l}$. Further, $\mathbf {s}_{d,l}$ and $\mathbf {A}_{d,l}$ can be normalized as $\mathbb {E}\left[ {\mathbf {s}_{d,l}}{\mathbf {s}}_{d,l}^{H}\right] =\mathbf {I}_{n}$ and $\mathrm {Tr}\left( {\mathbf {A}_{d,l}}{\mathbf {A}}_{d,l}^{H}\right) ={P_{tr}/{U}_{d}}$ for $l=1,2,\ldots ,{U}_{d}$, respectively. $P_{tr}$ is the maximum transmitted power at the base station and we assume that is equal for every user. Therefore, the vector that is precoded for $l^{th}$ user is represented by

2 $$\begin{aligned} \mathbf {x}_{d,l}=\mathbf {A}_{d,l}{\mathbf {s}}_{d,l}. \end{aligned}$$

By inserting (2), expression (1) can be rewritten as

3 $$\begin{aligned} \begin{aligned} {\mathbf {y}_{d,l}}&={\mathbf {H}}_{d,l}\mathbf {A}_{d,l}{\mathbf {s}}_{d,l}+{\mathbf {H}}_{d,l} \sum _{i\ne {l}}^{U_{d}}\mathbf {A}_{d,i}{\mathbf {s}}_{d,i}+\mathbf {H}_{cci,l}\mathbf {x}_{cci,l}+\mathbf {v}_{d,l},~{i,l}={1,2,\ldots ,{U_{d}}}. \end{aligned} \end{aligned}$$

As shown in the right-hand side of (3), the four parts designate the desired signal, MUI, CCI and noise, sequentially.

### Uplink signal model

For the UL channel, we also use a precoder matrix $\mathbf {B}_{u,l}{\in \mathscr {C}^{{{N}_{u,l}\times {m}}}},~l={1,2,\ldots ,{U_{u}}}$ for prior communication. The letter *m* stands for the number of streams per user and it is assumed to be $m\le {N}_{r}$. The precoder can be normalized as $\mathrm {Tr}\left( {\mathbf {B}_{u,l}}{\mathbf {B}}_{u,l}^{H}\right) ={P_{ul,l}}$ for $l=1,2,\ldots ,{U}_{u}$. The transmitted power at each user *l* is designated by $P_{ul,l}$ and assumes that the same for every user. Therefore, the precoded signal vector for the transmit signal $\mathbf {x}_{u,l}\in \mathscr {C}^{{N}_{u,l}\times {1}}$ for the $l^{th}$ user can be expressed as

4 $$\begin{aligned} \mathbf {x}_{u,l}=\mathbf {B}_{u,l}{\mathbf {z}}_{u,l} \end{aligned}$$

where $\mathbf {z}_{u,l}\in \mathscr {C}^{m\times {1}}$ denotes the intended signal vectors of user *l*. The input signal vectors are assumed to be independent with unit variance and it can be normalized as $\mathbb {E}\left[ {\mathbf {z}_{u,l}}{\mathbf {z}}_{u,l}^{H}\right] =\mathbf {I}_{m}$ for $l=1,2,\ldots ,{U}_{u}$.

At the Full-duplex based base station, the received signal vector $\mathbf {y}_{u}\in \mathscr {C}^{{N}_{r}\times {1}}$ can be given by

5 $$\begin{aligned} \mathbf {y}_{u}&=\sum _{l=1}^{U_{u}}{\mathbf {H}}_{u,l}{\mathbf {x}_{u,l}}+\mathbf {H}_{SI,l}\mathbf {x}_{SI,l}+\mathbf {v}_{u}\nonumber \nonumber \\&={\mathbf {H}}_{u,l}\mathbf {B}_{u,l}{\mathbf {z}}_{u,l}+\sum _{i\ne {l}}^{U_{u}}{\mathbf {H}}_{u,i}\mathbf {B}_{u,i}{\mathbf {z}}_{u,i}+\mathbf {H}_{SI,l}\mathbf {x}_{SI,l}+\mathbf {v}_{u} \end{aligned}$$

where ${\mathbf {H}}_{u,l}{\in \mathscr {C}^{{N}_{r}\times {N_{u,l}}}}$ stands for the Rician flat fading channel matrices of user *l*; $\mathbf {v}_{u}\in \mathscr {C}^{{N}_{r}\times {1}}$ represents AWGN distributed as $\mathscr{CN}\mathscr{}({0},{\sigma _{v}^2\mathbf {I}}_{{N}_{r}})$; $\mathbf {x}=\mathbf {x}_{SI,l}\in \mathscr {C}^{{N}_{t}\times {1}}$ and $\mathbf {H}_{SI,l}\in \mathscr {C}^{{N}_{r}\times {{N}_{t}}}$ are the transmitted vector and the channel matrix, respectively, of the self-interference because of the Full-duplex-based BS for user *l* in the uplink communication. On the right-hand side of (5), the four terms stand for the desired signal, Multi-user interference, self-interference, and noise of the $l\mathrm{th}$ user, respectively.

## Proposed SLNR precoding scheme and problem formulations

The problem formulations of the uplink and downlink channels are provided in the following subsequent subsections.

### Downlink channel model

This reconsiders (3) before we go forward to the optimization formulations. The interferences and noise in (3) are challenging to decode the desired signal vectors for a given user. Consequently, this study proposes an SLNR-based method that represses the impacts of CCI as well as maximizes the SE of the downlink Full-duplex Multi-user Multiple Input Multiple Output systems with the existence of CCI for a given receiver, based on only co-channel interference covariance matrix at the BS.

Thus, the $N_{d,l}\times N_{d,l}$ co-channel interference plus noise covariance matrices of (3) for the $l\mathrm{th}$ user is expressed as

6 $$\begin{aligned} \begin{aligned} {\mathbf {M}}_{int,l}&=\mathbf {H}_{cci,l} {\mathrm E}[\mathbf {x}_{cci,l}\mathbf {x}_{cci,l}^{H}]\mathbf {H}_{cci,l}^{H}+\sigma _{v}^2\mathbf {I}_{N_{d,l}}\\ {}&=\mathbf {H}_{cci,l}\mathbf {G}_{cci,l}\mathbf {H}_{cci,l}^{H}+\sigma _{v}^2\mathbf {I}_{N_{d,l}} \end{aligned} \end{aligned}$$

where $\mathbf {G}_{cci,l}=\mathrm {E}\left[ \mathbf {x}_{cci,l}\mathbf {x}_{cci,l}^{H}\right]$ stands for the covariance matrices of the co-channel interference as well $\mathrm {Tr}(\mathbf {G}_{cci,l})={P}_{cci,l}$. ${P}_{cci,l}$ is the transmitted power of the UL users. We use Principal Component Analysis (PCA) to whiten the interference signal. Then, the suppression matrix $\mathbf {Q}_{l}$ is obtained from the eigenvector decomposition (ED) of the co-channel interference plus noise covariance matrices and it can be represented as ${\mathbf {M}}_{int,l}={\mathbf {U}}_{l}\Sigma _{l}{\mathbf {U}}_{l}^{H}$. Therefore, the whitening matrix is given by $\mathbf {Q}_{l}=\Sigma _{l}^{-1/2}{\mathbf {U}}_{l}^{H}$. Expression (3) can be re-represented as follows by multiplying the whitening matrix $\mathbf {Q}_{l}$.

7 $$\begin{aligned} \mathbf {r}_{d,l}=\mathbf {Q}_{l}{\mathbf {H}}_{d,l}\mathbf {A}_{d,l}{\mathbf {s}}_{d,l}+\mathbf {Q}_{l}{\mathbf {H}}_{d,l} \sum _{i\ne {l}}^{U_{d}}\mathbf {A}_{d,i}{\mathbf {s}}_{d,i}+\varvec{\tilde{v}}_{d,l} \end{aligned}$$

where $\varvec{\tilde{v}}_{d,l}=\mathbf {Q}_{l}(\mathbf {H}_{cci,l}\mathbf {x}_{cci,l}+\mathbf {v}_{d,l})$. $\varvec{\tilde{v}}_{d,l}$ has a covariance of $\mathbf {I}_{N_{d,l}}$ due to the interference suppression matrix.

Now, let us see the decoded vector at the $l\mathrm{th}$ user for the matched filter ${\mathbf {W}}_{d,l}^{H}$ before we carry on to the precoder design and this can be given by

8 $$\begin{aligned} {\varvec{\hat{s}}}_{d,l}={\mathbf {W}}_{d,l}^{H}{\mathbf {r}}_{d,l}. \end{aligned}$$

The matched filter, ${\mathbf {W}}_{d,l}^{H}$, can be represented as

9 $$\begin{aligned} {\mathbf {W}}_{d,l}^{H}=\zeta \left( \mathbf {Q}_{l}{\mathbf {H}}_{d,l}\mathbf {A}_{d,l}\right) ^{H} \end{aligned}$$

where $\zeta$ designates the proportionality constant. Now, inserting (7) and (9) into (8), the decoded signal vector is denoted by

10 $$\begin{aligned} \begin{aligned} {\varvec{\hat{s}}}_{d,l}&=\zeta {\mathbf {A}}_{d,l}^{H}{\mathbf {H}}_{d,l}^{H}\mathbf {Q}_{l}^{H}\mathbf {Q}_{l}{\mathbf {H}}_{d,l}{\mathbf {A}}_{d,l}{\mathbf {s}}_{d,l}+\zeta \left( {\mathbf {A}}_{d,l}^{H}{\mathbf {H}}_{d,l}^{H}\mathbf {Q}_{l}^{H}\right) \times \left( \mathbf {Q}_{l}{\mathbf {H}}_{d,l}\sum _{i\ne {l}}^{U_{d}}{\mathbf {A}}_{d,i}{\mathbf {s}}_{d,i}+\varvec{\tilde{v}}_{d,l}\right) . \end{aligned} \end{aligned}$$

For the sake of decoupling the multi-streams, re-expressing (10) and we can get additional design constraints as described below.

11 $$\begin{aligned} {\varvec{\hat{s}}}_{d,l}={\mathbf {D}}_{l}^{'}{\mathbf {s}}_{d,l}+\zeta \left( {\mathbf {A}}_{d,l}^{H}{\mathbf {H}}_{d,l}^{H}\mathbf {Q}_{l}^{H}\right) \left( \mathbf {Q}_{l}{\mathbf {H}}_{d,l}\sum _{i\ne {l}}^{U_{d}}{\mathbf {A}}_{d,i}{\mathbf {s}}_{d,i}+\varvec{\tilde{v}}_{d,l}\right) . \end{aligned}$$

The symbol ${\mathbf {D}}_{l}^{'}$ represents several diagonal matrices and the additional design limitation can be expressed as

12 $$\begin{aligned} {\mathbf {A}}_{d,l}^{H}{\mathbf {H}}_{d,l}^{H}\mathbf {Q}_{l}^{H}\mathbf {Q}_{l}{\mathbf {H}}_{d,l}{\mathbf {A}}_{d,l}={\mathbf {D}}_{l}^{'}. \end{aligned}$$

Here, the problem formulations of the precoder design by making use of maximizing the SINR metric for the $l\mathrm{th}$ user is given by

13 $$\begin{aligned} \mathrm {SINR}_{l}^{d}=\frac{{\mathrm Tr}\left( {\mathbf {A}}_{d,l}^{H}{\mathbf {H}}_{d,l}^{H}\mathbf {Q}_{l}^{H}\mathbb {E}\left[ {\mathbf {s}}_{d,l}{\mathbf {s}}_{d,l}^{H}\right] \mathbf {Q}_{l}{\mathbf {H}}_{d,l}{\mathbf {A}}_{d,l}\right) }{{N_{d,l}}\sigma _{\tilde{v}}^2+\sum _{i\ne {l}}^{U_{d}}{\mathrm Tr}\left( {\mathbf {A}}_{d,i}^{H}{\mathbf {H}}_{d,l}^{H}\mathbf {Q}_{l}^{H}\mathbb {E}\left[ {\mathbf {s}}_{d,i}{\mathbf {s}}_{d,i}^{H}\right] \mathbf {Q}_{l}{\mathbf {H}}_{d,l}{\mathbf {A}}_{d,i}\right) }=\frac{{\mathrm Tr}\left( {\mathbf {A}}_{d,l}^{H}{\mathbf {H}}_{d,l}^{H}\mathbf {Q}_{l}^{H}\mathbf {Q}_{l}{\mathbf {H}}_{d,l}{\mathbf {A}}_{d,l}\right) }{{N_{d,l}}\sigma _{\tilde{v}}^2+\sum _{i\ne {l}}^{U_{d}}{\mathrm Tr}\left( {\mathbf {A}}_{d,i}^{H}{\mathbf {H}}_{d,l}^{H}\mathbf {Q}_{l}^{H}\mathbf {Q}_{l}{\mathbf {H}}_{d,l}{\mathbf {A}}_{d,i}\right) } \end{aligned}$$

where $\sigma _{\tilde{v}}^2=1$ due to the interference suppression matrix. The precoder design based on (13) criterion arises in a coupled coefficients of $\left\{ \mathbf {A}_{d,l}\right\} _{l}^{U_d}$ for $l=1,2,\ldots ,U_d$. We use the concept of signal leakage, the SLNR as an optimization criterion in^28–30^ due to the fact that SINR in (13) has no closed-form solutions^26,39^. Leakage refers to the amount of signal power leaking from one user to another. The SLNR metric can optimize a coupled optimization problem into an easily solvable, fully decoupled problem.

Thus, the optimization problem based on the SLNR criterion for the $l\mathrm{th}$ user is given by

14 $$\begin{aligned} \begin{aligned} \mathrm {SLNR}_{l}^{d}&=\frac{{\mathrm Tr}\left( {\mathbf {A}}_{d,l}^{H}{\mathbf {H}}_{d,l}^{H}\mathbf {Q}_{l}^{H}\mathbf {Q}_{l}{\mathbf {H}}_{d,l}{\mathbf {A}}_{d,l}\right) }{{N_{d,l}}\sigma _{\tilde{v}}^2+\sum _{i\ne {l}}^{U_{d}}{\mathrm Tr}\left( {\mathbf {A}}_{d,l}^{H}{\mathbf {H}}_{d,i}^{H}\mathbf {Q}_{l}^{H}\mathbf {Q}_{l}{\mathbf {H}}_{d,i}{\mathbf {A}}_{d,l}\right) }\\ {}&=\frac{{\mathrm Tr} \left( {\mathbf {A}}_{d,l}^{H}{\mathbf {H}}_{d,l}^{H}\mathbf {Q}_{l}^{H}\mathbf {Q}_{l}{\mathbf {H}}_{d,l}{\mathbf {A}}_{d,l}\right) }{{\mathrm Tr}\left( {\mathbf {A}}_{d,l}^{H}\left( {\left( {N_{d,l}}{U_{d}}/P_{tr}\right) \mathbf {I}_{N_{t}}+{\varvec{\hat{H}}}_{d,l}^{H}\mathbf {Q}_{l}^{H}\mathbf {Q}_{l}{\varvec{\hat{H}}}_{d,l}}\right) {\mathbf {A}}_{d,l}\right) } \end{aligned} \end{aligned}$$

where the spanned of ${\varvec{\hat{H}}}_{d,l}$ without ${\mathbf {H}}_{d,l}$ only is

15 $$\begin{aligned} \begin{aligned} {\varvec{\hat{H}}}_{d,l}=\left[ {\mathbf {H}}_{d,1}^{H}\cdots {\mathbf {H}}_{d,l-1}^{H}{\mathbf {H}}_{d,l+1}^{H}\cdots {\mathbf {H}}_{d,U_{d}}^{H}\right] ^{H}. \end{aligned} \end{aligned}$$

Now, the beamforming coefficient vectors can be found, by maximizing (14) subject to $\mathrm {Tr}\left( {\mathbf {A}_{d,l}}{\mathbf {A}}_{d,l}^{H}\right) ={P_{tr}/{U}_{d}}$ and (12) for $l={1,2,\ldots ,{U_{d}}}$ as can be expressed in (16).

16 $$\begin{aligned} \begin{aligned}{}&{\mathbf {A}}_{d,l}^{o}=\arg \max \limits _{{\mathbf {A}}_{d,l}\in {\mathscr C}^{{{N}_{d,l}\times {n}}}}\frac{{\mathrm Tr}\left( {\mathbf {A}}_{d,l}^{H}{\mathbf {H}}_{d,l}^{H}\mathbf {Q}_{l}^{H}\mathbf {Q}_{l}{\mathbf {H}}_{d,l}{\mathbf {A}}_{d,l}\right) }{{\mathrm Tr}\left( {\mathbf {A}}_{d,l}^{H}\left( \left( {N_{d,l}{U_{d}}}/P_{tr}\right) \mathbf {I}_{N_{t}}+{\varvec{\hat{H}}}_{d,l}^{H}\mathbf {Q}_{l}^{H}\mathbf {Q}_{l}{\varvec{\hat{H}}}_{d,l}\right) {\mathbf {A}}_{d,l}\right) }\\ {}&{{\mathrm s.t.}} \quad {\mathrm Tr}\left( {\mathbf {A}}_{d,l}{\mathbf {A}}_{d,l}^{H}\right) =P_{tr}/{U_{d}} \quad \text {and}\quad \ {\mathbf {A}}_{d,l}^{H}{\mathbf {H}}_{d,l}^{H}\mathbf {Q}_{l}^{H}\mathbf {Q}_{l}{\mathbf {H}}_{d,l}{\mathbf {A}}_{d,l}={\mathbf {D}}_{l}^{\prime},\quad l={1,2,\ldots ,{U_d}}. \end{aligned} \end{aligned}$$

Since, the pair $\{{\mathbf {H}}_{d,l}^{H}\mathbf {Q}_{l}^{H}\mathbf {Q}_{l}{\mathbf {H}}_{d,l},\left( {N_{d,l}}{U_{d}}/P_{tr}\right) \mathbf {I}_{N_{t}}+{\varvec{\hat{H}}}_{d,l}^{H}\mathbf {Q}_{l}^{H}\mathbf {Q}_{l}{\varvec{\hat{H}}}_{d,l}\}$ in (16) are Hermitian-Positive-Semi-definite (HPSD) and Hermitian-positive-definite (HPD) matrices, respectively, there exists a non-singular ${\mathbf {J}}_{l}$ $({N_t}\times {N_t})$ matrix by making use of general eigenvalue decomposition (GEVD) in^40^ such that

17 $$\begin{aligned} \begin{aligned}{}&{\mathbf {J}}_{l}^{H}{\mathbf {H}}_{d,l}^{H}\mathbf {Q}_{l}^{H}\mathbf {Q}_{l}{\mathbf {H}}_{d,l}{\mathbf {J}}_{l}=\Phi _{N_{t}}={\rm diag}\left( \rho _{l,1},\rho _{l,2},\ldots ,\rho _{l,N_{t}}\right) \\ {}&{\mathbf {J}}_{l}^{H}\left( \left( {N_{d,l}}{U_{d}}/P_{tr}\right) \mathbf {I}_{N_{t}}+{\varvec{\hat{H}}}_{d,l}^{H}\mathbf {Q}_{l}^{H}\mathbf {Q}_{l}{\varvec{\hat{H}}}_{d,l}\right) {\mathbf {J}}_{l}=\mathbf {I}_{N_{t}}\\ \end{aligned} \end{aligned}$$

where $\rho _{l,1}\ge \rho _{l,2}\ge \cdots \ge \rho _{l,n} \ge \cdots \ge \rho _{l,N_{d,l}}$ $>{0},\rho _{{l,N_{d,l}}+1}=\cdots =\rho _{l,N_{t}}=0$. For the pair matrices $\left\{ {\mathbf {H}}_{d,l}^{H}\mathbf {Q}_{l}^{H}\mathbf {Q}_{l}{\mathbf {H}}_{d,l},\left( {N_{d,l}}{U_{d}}/P_{tr}\right) \mathbf {I}_{N_{t}}+{\varvec{\hat{H}}}_{d,l}^{H}\mathbf {Q}_{l}^{H}\mathbf {Q}_{l}{\varvec{\hat{H}}}_{d,l}\right\}$, the eigenvalues and eigenvectors are given by $\rho _{l,1},\rho _{l,2},\ldots ,\rho _{l,N_{t}}$ and the columns of ${\mathbf {J}}_{l}$, respectively.

Now, we can define the precoder as ${\mathbf {A}}_{l}={\mathbf {J}}_{l}{\mathbf {F}}_{l}$, where ${\mathbf {F}}_{l}$ is $({N_t}\times {n})$ matrix. Since the matrix ${\mathbf {J}}_{l}$ is invertible, there is a one-to-one correspondence between ${\mathbf {A}}_{l}$ and ${\mathbf {F}}_{l}$. Then, inserting it into (16) becomes

18 $$\begin{aligned} \begin{aligned}{}&\frac{ {\rm Tr}\left( {\mathbf {A}}_{d,l}^{H}{\mathbf {H}}_{d,l}^{H}\mathbf {Q}_{l}^{H}\mathbf {Q}_{l}{\mathbf {H}}_{d,l}{\mathbf {A}}_{d,l}\right) }{ {\rm Tr}\left( {\mathbf {A}}_{d,l}^{H}\left( \left( {N_{d,l}}{U_{d}}/P_{tr}\right) \mathbf {I}_{N_{t}}+{\varvec{\hat{H}}}_{d,l}^{H}\mathbf {Q}_{l}^{H}\mathbf {Q}_{l}{\varvec{\hat{H}}}_{d,l}\right) {\mathbf {A}}_{d,l}\right) }\\ {}&=\frac{ {\rm Tr}\left( {\mathbf {F}}_{l}^{H}{\mathbf {J}}_{l}^{H}{\mathbf {H}}_{d,l}^{H}\mathbf {Q}_{l}^{H}\mathbf {Q}_{l}{\mathbf {H}}_{d,l}{\mathbf {J}}_{l}{\mathbf {F}}_{l}\right) }{{\mathrm Tr}\left( {\mathbf {F}}_{l}^{H}{\mathbf {J}}_{l}^{H}\left( \left( {N_{d,l}}{U_{d}}/P_{tr}\right) \mathbf {I}_{N_{t}}+{\varvec{\hat{H}}}_{d,l}^{H}\mathbf {Q}_{l}^{H}\mathbf {Q}_{l}{\varvec{\hat{H}}}_{d,l}\right) {\mathbf {J}}_{l}{\mathbf {F}}_{l}\right) }\\ {}&=\frac{{\mathrm Tr}\left( {\mathbf {F}}_{l}^{H}\Phi _{N_{t}}{\mathbf {F}}_{l}\right) }{{\mathrm Tr}\left( {\mathbf {F}}_{l}^{H}{\mathbf {F}}_{l}\right) }. \end{aligned} \end{aligned}$$

The preferred precoding matrix, ${\mathbf {A}}_{l}$, which maximizes (18) can be given by $\left[ \mathbf {I}_{n};\mathbf {0}\right]$. The preferences also satisfy the constraint matrices in (16), ${\mathbf {A}}_{d,l}^{H}{\mathbf {H}}_{d,l}^{H}\mathbf {Q}_{l}^{H}\mathbf {Q}_{l}{\mathbf {H}}_{d,l}{\mathbf {A}}_{d,l}$ is diagonal as we needed. The simplified maximum value of (18) can be written by

19 $$\begin{aligned} {{\mathrm SLNR}}_{l}^{d,max}=1/n{\sum _{i=1}^{n}\rho _{l,i}}. \end{aligned}$$

Consequently, the optimum precoding coefficients for the user *l* in (16) can be given by

20 $$\begin{aligned} {\mathbf {A}}_{d,l}^{o}=\xi {\mathbf {J}}_{l}{\mathbf {F}}_{l}. \end{aligned}$$

The scaling factor $\xi$ is used to yield ${\mathrm Tr}({\mathbf {A}}_{d,l}{\mathbf {A}}_{d,l}^{H})=P_{tr}/{U_{d}}$.

The total sum rate of the downlink communication using (20) is achieved by

21 $$\begin{aligned} \begin{aligned} {{\mathrm R}}_{d}&=\sum _{l=1}^{U_d}\log _{2}\left| \mathbf {I}_{N_{d,l}}+\left( \mathbf {Q}_{l}{\mathbf {H}}_{d,l}{\mathbf {A}}_{d,l}^{o}{\mathbf {A}}_{d,l}^{o,H}{\mathbf {H}}_{d,l}^{H}\mathbf {Q}_{l}^{H}\right) \times \left( \sigma _{\tilde{v}}^{2}\mathbf {I}_{N_{d,l}} +\sum _{i\ne {l}}^{U_{d}}{\mathbf {Q}_{l}}{\mathbf {H}}_{d,l}{\mathbf {A}}_{d,i}^{o}{\mathbf {A}}_{d,i}^{o,H}{\mathbf {H}}_{d,l}^{H}\mathbf {Q}_{l}^{H}\right) ^{-1}\right| . \end{aligned} \end{aligned}$$

Now, we can observe the effects of the Rician fading environment in (21) of the DL channel, and it can be described below.

*Result 1:* Let us take ${\mathbf {H}}_{d,l}$ be Rician flat fading channel. Using the relation $\left| \mathbf {I}_{q}+\mathbf{{M}}_{q\times {r}}{} \mathbf{{N}}_{r\times {q}}\right| =\left| \mathbf {I}_{r}+\mathbf{{M}}_{r\times {q}}{} \mathbf{{N}}_{q\times {r}}\right|$ and for a given transmitted power, the expression (21) becomes

22 $$\begin{aligned} \begin{aligned} {{\mathrm R}}_{d}&=\sum _{l=1}^{U_d}\log _{2}|\mathbf {I}_{N_{d,l}}+{\frac{{1}}{K+1}}\times \mathbf{{S}}_{d}\mathbf {Q}_{l}{\mathbf {D}}_{d}{\mathbf {A}}_{d,l}^{o}{\mathbf {A}}_{d,l}^{o,H}{\mathbf {D}}_{d}^{H}\mathbf {Q}_{l}^{H}{} \mathbf{{S}}_{d}^{H}| \end{aligned} \end{aligned}$$

where $$\left( \sigma _{\tilde{v}}^{2}\mathbf {I}_{N_{d,l}} +\sum _{i\ne {l}}^{U_{d}}{\mathbf {Q}_{l}}{\mathbf {H}}_{d,l}{\mathbf {A}}_{d,i}^{o}{\mathbf {A}}_{d,i}^{o,H}{\mathbf {H}}_{d,l}^{H}\mathbf {Q}_{l}^{H}\right) ^{-1}=\mathbf{{S}}_{d}^{H}{} \mathbf{{S}}_{d}$$ and $$\mathbf{{D}}_{d}=\sqrt{K+1}{\mathbf {H}}_{d,l}=\sqrt{K}{\bar{\mathbf {H}}_{d,l}}+{\tilde{\mathbf {H}}_{d,l}}\sim ~\mathscr{CN}\mathscr{}\left( \sqrt{K}\bar{\mathbf {H}}_{d,l},{\sigma _{\tilde{v}}^2}{\mathbf {I}}_{{N}_{d,l}}\right)$$.

For a fixed power transmitter, the higher *K* (Rician factor) implies a lower multi-path attachment results, the smaller the channel capacity.

### Uplink signal model

As expressed in (5), the desired signal is difficult to decode this is because of the noise and interferences. Hence, we propose a precoding design based on the SLNR scheme. This scheme whitens the impact of self-interference and maximizes the spectral efficiency of the UL Full-duplex Multi-user MIMO wireless communication in the existence of self-interference for a given receiver, by making use of only the self-interference covariance matrices at the transmitting side.

Therefore, the $N_r\times {N_r}$ self-interference plus noise covariance matrix in (5) for the $l{\rm th}$ user can be expressed as

23 $$\begin{aligned} \begin{aligned} {\mathbf {O}}_{int,l}&=\mathbf {H}_{SI,l}{\mathrm E}\left[ \mathbf {x}_{SI,l}\mathbf {x}_{SI,l}^{H}\right] \mathbf {H}_{SI,l}^{H}+\sigma _{v}^2\mathbf {I}_{N_r}\\ {}&=\mathbf {H}_{SI,l}\mathbf {C}_{SI,l}\mathbf {H}_{SI,l}^{H}+\sigma _{v}^2\mathbf {I}_{N_r} \end{aligned} \end{aligned}$$

where $\mathbf {C}_{SI,l}=\mathrm {E}\left[ \mathbf {x}_{SI,l}\mathbf {x}_{SI,l}^{H}\right]$ denotes covariance matrix of the self-interference and ${\mathrm Tr}\left( \mathbf {C}_{SI,l}\right) ={P}_{SI,l}$. The maximum transmitted power at the base station is represented ${P}_{SI,l}$. To whiten the interference signal, we propose a PCA suppression matrix. The whitening matrix $\mathbf {T}_{l}$ is obtained by the eigenvector decomposition of the self-interference plus noise covariance matrices of ${\mathbf {O}}_{int,l}={\mathbf {L}}_{l}\Gamma _{l}{\mathbf {L}}_{l}^{H}$. Therefore, the suppression matrix is described by $\mathbf {T}_{l}=\Gamma _{l}^{-1/2}{\mathbf {L}}_{l}^{H}$. Then multiplying (5) by $\mathbf {T}_{l}$ and we can get

24 $$\begin{aligned} \mathbf {r}_{u,l}&=\mathbf {T}_{l}\mathbf {H}_{u,l}\mathbf {B}_{u,l}{\mathbf {z}}_{u,l} +\mathbf {T}_{l}\sum _{i\ne {l}}^{U_{u}}{\mathbf {H}}_{u,i}\mathbf {B}_{u,i}{\mathbf {z}}_{u,i}+\varvec{\tilde{v}}_{u,l} \end{aligned}$$

where $\varvec{\tilde{v}}_{u,l}=\mathbf {T}_{l}(\mathbf {H}_{SI,l}\mathbf {x}_{SI,l}+\mathbf {v}_{u,l})$. The covariance matrix of $\varvec{\tilde{v}}_{u,l}$ is an identity matrix due to the interference suppression matrix.

For a matched filter ${\mathbf {W}}_{u,l}^{H}$, the decoded vector of the $l\mathrm{th}$ user is written as

25 $$\begin{aligned} \begin{aligned} {\varvec{\hat{z}}}_{u,l}&={\mathbf {W}}_{u,l}^{H}{\mathbf {r}}_{u,l}. \end{aligned} \end{aligned}$$

The matched filter, ${\mathbf {W}}_{u,l}^{H}$, can be expressed as

26 $$\begin{aligned} {\mathbf {W}}_{u,l}^{H}=\chi \left( \mathbf {T}_{l}{\mathbf {H}}_{u,l}\mathbf {B}_{u,l}\right) ^{H} \end{aligned}$$

where $\chi$ is the proportionality constant. Inserting (24) and (26) into (25), the decoded signal becomes

27 $$\begin{aligned} \begin{aligned} {\varvec{\hat{z}}}_{u,l}&=\chi {\mathbf {B}}_{u,l}^{H}{\mathbf {H}}_{u,l}^{H}\mathbf {T}_{l}^{H}\mathbf {T}_{l}{\mathbf {H}}_{u,l}{\mathbf {B}}_{u,l}{\mathbf {z}}_{u,l}+\chi \left( {\mathbf {B}}_{u,l}^{H}{\mathbf {H}}_{u,l}^{H}\mathbf {T}_{l}^{H}\right) \left( \mathbf {T}_{l}\sum _{i\ne {l}}^{U_{u}}{\mathbf {H}}_{u,i}\mathbf {B}_{u,i}{\mathbf {z}}_{u,i}+\varvec{\tilde{v}}_{u,l}\right) . \end{aligned} \end{aligned}$$

Re-expressing (27) to decouple the multi-streams and again to give an additional design constraint as

28 $$\begin{aligned} \begin{aligned} {\varvec{\hat{z}}}_{u,l}&={\mathbf {D}}_{u}^{'}{\mathbf {z}}_{u,l}+\chi \left( {\mathbf {B}}_{u,l}^{H}{\mathbf {H}}_{u,l}^{H}\mathbf {T}_{l}^{H}\right) \times \left( \mathbf {T}_{l}\sum _{i\ne {l}}^{U_{u}}{\mathbf {H}}_{u,i}\mathbf {B}_{u,i}{\mathbf {z}}_{u,i}+\varvec{\tilde{v}}_{u,l}\right) \end{aligned} \end{aligned}$$

where ${\mathbf {D}}_{u}^{'}$ represents some diagonal matrix. Now, the design constraints can be represented as

29 $$\begin{aligned} {\mathbf {B}}_{u,l}^{H}{\mathbf {H}}_{u,l}^{H}\mathbf {T}_{l}^{H}\mathbf {T}_{l}{\mathbf {H}}_{u,l}{\mathbf {B}}_{u,l}={\mathbf {D}}_{u}^{'}. \end{aligned}$$

Next, designing the precoder coefficients by making use of maximization of SINR at the $l^{th}$ user is formulated by

30 $$\begin{aligned} \begin{aligned} \mathrm {SINR}_{l}^{u}=\frac{{\mathrm Tr}\left( {\mathbf {B}}_{u,l}^{H}{\mathbf {H}}_{u,l}^{H}\mathbf {T}_{l}^{H}\mathbf {T}_{l}{\mathbf {H}}_{u,l}{\mathbf {B}}_{u,l}\right) }{{N_{r}}\sigma _{\tilde{v}}^2+\sum _{i\ne {l}}^{U_{u}}{\mathrm Tr}\left( {\mathbf {B}}_{u,i}^{H}{\mathbf {H}}_{u,i}^{H}\mathbf {T}_{l}^{H}\mathbf {T}_{l}{\mathbf {H}}_{u,i}{\mathbf {B}}_{u,i}\right) } \end{aligned} \end{aligned}$$

where $\sigma _{\tilde{v}}^2=1$ due to the suppression matrix. Designing the precoder based on the above metric rises in a problem of $U_u$ coupled coefficients of $\{\mathbf {B}_{u,l}\}_{l}^{U_u}$ for $l=1,2,\ldots ,U_u$ and this indicate that it has no closed form solutions^26,39^. Therefore, we employ another criterion called SLNR-precoding scheme^28–30^.

The SLNR of the $l\mathrm{th}$ user based on the SLNR criterion can be represented by

31 $$\begin{aligned} \begin{aligned} \mathrm {SLNR}_{l}^{u}&=\frac{{\mathrm Tr}\left( {\mathbf {B}}_{u,l}^{H}{\mathbf {H}}_{u,l}^{H}\mathbf {T}_{l}^{H}\mathbf {T}_{l}{\mathbf {H}}_{u,l}{\mathbf {B}}_{u,l}\right) }{{N_{r}}\sigma _{\tilde{v}}^2+{\mathrm Tr}\left( \sum _{i\ne {l}}^{U_{u}}{\mathbf {B}}_{u,l}^{H}{\mathbf {H}}_{u,i}^{H}\mathbf {T}_{l}^{H}\mathbf {T}_{l}{\mathbf {H}}_{u,i}{\mathbf {B}}_{u,l}\right) }\\ {}&=\frac{{\mathrm Tr}\left( {\mathbf {B}}_{u,l}^{H}{\mathbf {H}}_{u,l}^{H}\mathbf {T}_{l}^{H}\mathbf {T}_{l}{\mathbf {H}}_{u,l}{\mathbf {B}}_{u,l}\right) }{{\mathrm Tr}\left( {\mathbf {B}}_{u,l}^{H} \left( \left( {N_{r}}/P_{ul,l}\right) \mathbf {I}_{N_{u,l}}+{\varvec{\hat{H}}}_{u,l}^{H}\mathbf {T}_{l}^{H}\mathbf {T}_{l}{\varvec{\hat{H}}}_{u,l}\right) {\mathbf {B}}_{u,l}\right) } \end{aligned} \end{aligned}$$

where the spanned of ${\varvec{\hat{H}}}_{u,l}$ without ${\mathbf {H}}_{u,l}$ can be expressed as

32 $$\begin{aligned} \begin{aligned} {\varvec{\hat{H}}}_{u,l}=\left[ {\mathbf {H}}_{u,1}^{H}\cdots {\mathbf {H}}_{u,l-1}^{H}{\mathbf {H}}_{u,l+1}^{H}\cdots {\mathbf {H}}_{u,U_{u}}^{H}\right] ^{H}. \end{aligned} \end{aligned}$$

Thus, the precoding coefficient vectors can be obtained, by maximizing (31) subject to ${\mathrm Tr}\left( {\mathbf {B}_{u,l}}{\mathbf {B}}_{u,l}^{H}\right) =P_{ul,l}$ and (29) for $l={1,2,\ldots ,{U_{d}}}$ as given as in (33).

33 $$\begin{aligned} \begin{aligned}{}&{\mathbf {B}}_{u,l}^{o}=\arg \max \limits _{{\mathbf {B}}_{u,l}\in {\mathscr C}^{{{N}_{u,l}\times {m}}}}\frac{ {\rm Tr}\left( {\mathbf {B}}_{u,l}^{H}{\mathbf {H}}_{u,l}^{H}\mathbf {T}_{l}^{H}\mathbf {T}_{l}{\mathbf {H}}_{u,l}{\mathbf {B}}_{u,l}\right) }{ {\mathrm Tr}\left( {\mathbf {B}}_{u,l}^{H}\left( \left( {N_{r}}/P_{ul,l}\right) \mathbf {I}_{N_{u,l}}+{\varvec{\hat{H}}}_{u,l}^{H}\mathbf {T}_{l}^{H}\mathbf {T}_{l}{\varvec{\hat{H}}}_{u,l}\right) {\mathbf {B}}_{u,l}\right) }\\&{{\mathrm s.t.}} \quad {\mathrm Tr}\left( {\mathbf {B}}_{u,l}{\mathbf {B}}_{u,l}^{H}\right) =P_{ul,l} \quad \text {and}\quad \ {\mathbf {B}}_{u,l}^{H}{\mathbf {H}}_{u,l}^{H}\mathbf {T}_{l}^{H}\mathbf {T}_{l}{\mathbf {H}}_{u,l}{\mathbf {B}}_{u,l}={\mathbf {D}}_{u}^{\prime},\quad l={1,2,\ldots ,{U_u}}. \end{aligned} \end{aligned}$$

The pair matrices $\{{\mathbf {H}}_{u,l}^{H}\mathbf {T}_{l}^{H}\mathbf {T}_{l}{\mathbf {H}}_{u,l},\left( {N_{r}}/P_{ul,l}\right) \mathbf {I}_{N_{u,l}}+{\varvec{\hat{H}}}_{u,l}^{H}\mathbf {T}_{l}^{H}\mathbf {T}_{l}{\varvec{\hat{H}}}_{u,l}\}$ in (33) by definition are HPSD and HPD, respectively. Based on GEVD in^40^, there should exist a non-singular matrix ${\mathbf {P}}_{l}$ $({N_{u,l}}\times {N_{u,l}})$ such that

34 $$\begin{aligned} \begin{aligned}{}&{\mathbf {P}}_{l}^{H}{\mathbf {H}}_{u,l}^{H}\mathbf {T}_{l}^{H}\mathbf {T}_{l}{\mathbf {H}}_{u,l}{\mathbf {P}}_{l}=\Upsilon _{N_{u,l}}={\mathrm diag}\left( \gamma _{l,1},\gamma _{l,2},\ldots ,\gamma _{l,N_{u,l}}\right) \\ {}&{\mathbf {P}}_{l}^{H}\left( \left( {N_{r}}/P_{ul,l}\right) \mathbf {I}_{N_{u,l}}+{\varvec{\hat{H}}}_{u,l}^{H}\mathbf {T}_{l}^{H}\mathbf {T}_{l}{\varvec{\hat{H}}}_{u,l}\right) {\mathbf {P}}_{l}=\mathbf {I}_{N_{u,l}}\\ \end{aligned} \end{aligned}$$

where $\gamma _{l,1}\ge \gamma _{l,2}\ge \cdots \ge \gamma _{l,m} \ge \cdots \ge \gamma _{l,N_{u,l}}$ $\ge {0}$. For the generalized eigenspace pair matrices $\{{\mathbf {H}}_{u,l}^{H}\mathbf {T}_{l}^{H}\mathbf {T}_{l}{\mathbf {H}}_{u,l},({N_{r}}/P_{ul,l})\mathbf {I}_{N_{u,l}}+{\varvec{\hat{H}}}_{u,l}^{H}\mathbf {T}_{l}^{H}\mathbf {T}_{l}{\varvec{\hat{H}}}_{u,l}\}$, the columns of ${\mathbf {P}}_{l}$ and ${\mathrm diag}(\gamma _{l,1},\gamma _{l,2},\ldots ,\gamma _{l,N_{u,l}})$ are generalized eigenvectors and eigenvalues, sequentially^28,30^.

The precoder can be defined by ${\mathbf {B}}_{u,l}={\mathbf {P}}_{l}{\mathbf {G}}_{l}$, and ${\mathbf {G}}_{l}$ is given by the matrix $({N_{u,l}}\times {m})$. Due to the fact that ${\mathbf {P}}_{l}$ is invertible, there is a one-to-one correspondence between ${\mathbf {B}}_{u,l}$ and ${\mathbf {G}}_{l}$. Now, insert this into (33) gives

35 $$\begin{aligned} \begin{aligned}{}&\frac{{\mathrm Tr}\left( {\mathbf {B}}_{u,l}^{H}{\mathbf {H}}_{u,l}^{H}\mathbf {T}_{l}^{H}\mathbf {T}_{l}{\mathbf {H}}_{u,l}{\mathbf {B}}_{u,l}\right) }{{\mathrm Tr}\left( {\mathbf {B}}_{u,l}^{H}\left( \left( {N_{r}}/P_{ul,l}\right) \mathbf {I}_{N_{u,l}}+{\varvec{\hat{H}}}_{u,l}^{H}\mathbf {T}_{l}^{H}\mathbf {T}_{l}{\varvec{\hat{H}}}_{u,l}\right) {\mathbf {B}}_{u,l}\right) }\\ {}&=\frac{{\mathrm Tr}\left( {\mathbf {G}}_{l}^{H}{\mathbf {P}}_{l}^{H}{\mathbf {H}}_{u,l}^{H}\mathbf {T}_{l}^{H}\mathbf {T}_{l}{\mathbf {H}}_{u,l}{\mathbf {P}}_{l}{\mathbf {G}}_{l}\right) }{{\mathrm Tr}\left( {\mathbf {G}}_{l}^{H}{\mathbf {P}}_{l}^{H}\left( \left( {N_{r}}/P_{ul,l}\right) \mathbf {I}_{N_{u,l}}+{\varvec{\hat{H}}}_{u,l}^{H}\mathbf {T}_{l}^{H}\mathbf {T}_{l}{\varvec{\hat{H}}}_{u,l}\right) {\mathbf {P}}_{l}{\mathbf {G}}_{l}\right) }\\ {}&=\frac{ {\mathrm Tr}\left( {\mathbf {G}}_{l}^{H}\Upsilon _{N_{u,l}}{\mathbf {G}}_{l}\right) }{{\mathrm Tr}\left( {\mathbf {G}}_{l}^{H}{\mathbf {G}}_{l}\right) }. \end{aligned} \end{aligned}$$

To find the optimal precoder that maximizes the objective function (35) we can extract the leading *m* columns from ${\mathbf {P}}_{l}$ and is given by $\left[ \mathbf {I}_{m};\mathbf {0}\right]$. Besides, the choices that fulfill the constraint matrices in (33), ${\mathbf {B}}_{u,l}^{H}{\mathbf {H}}_{u,l}^{H}\mathbf {T}_{l}^{H}\mathbf {T}_{l}{\mathbf {H}}_{u,l}{\mathbf {B}}_{u,l}$ is diagonal as we wanted. Then, the resulting maximum value of (35) can be given as

36 $$\begin{aligned} \begin{aligned} {\mathrm {SLNR}}_{l}^{u,max}=1/m{\sum _{i=1}^{m}\gamma _{l,i}}. \end{aligned} \end{aligned}$$

Hence, the optimal precoder coefficients for the $l\mathrm{th}$ user in (33) becomes

37 $$\begin{aligned} {\mathbf {B}}_{u,l}^{o}=\beta {\mathbf {P}}_{l}{\mathbf {G}}_{l}. \end{aligned}$$

The scaling factor $\beta$ is used to give ${\mathrm Tr}\left( {\mathbf {B}}_{u,l}{\mathbf {B}}_{u,l}^{H}\right) =P_{ul,l}$.

The total sum rate of the uplink system is written by

38 $$\begin{aligned} \begin{aligned} {\mathrm {R}}_{u}&=\sum _{l=1}^{U_u}\log _{2}\left| \mathbf {I}_{N_{u,l}}+\left( \mathbf {T}_{l}{\mathbf {H}}_{u,l}{\mathbf {B}}_{u,l}^{o}{\mathbf {B}}_{u,l}^{o,H}{\mathbf {H}}_{u,l}^{H}\mathbf {T}_{l}^{H}\right) \times \left( \sigma _{\tilde{v}}^2\mathbf {I}_{N_{u,l}} +\sum _{i\ne {l}}^{U_{u}}\mathbf {T}_{l}{\mathbf {H}}_{u,i}{\mathbf {B}}_{u,i}^{o}{\mathbf {B}}_{u,i}^{o,H}{\mathbf {H}}_{u,i}^{H}\mathbf {T}_{l}^{H}\right) ^{-1}\right| . \end{aligned} \end{aligned}$$

The following result shows the effects of the Rician fading environment on the rate of communication of the UL channel.

*Result 2:* Consider ${\mathbf {H}}_{u,l}$ is the Rician fading environment, for a fixed power transmitted values plus based on the expression $\left| \mathbf {I}_{i}+\mathbf{{X}}_{i\times {k}}{} \mathbf{{Y}}_{k\times {i}}\right| =\left| \mathbf {I}_{k}+\mathbf{{X}}_{k\times {i}}{} \mathbf{{Y}}_{i\times {k}}\right|$, (38) can be represented as

39 $$\begin{aligned} \begin{aligned} {\mathrm {R}}_{u}&=\sum _{l=1}^{U_u}\log _{2}\left| \mathbf {I}_{N_{u,l}}+\mathbf{{S}}_{u}\mathbf {T}_{l}{\mathbf {H}}_{u,l}{\mathbf {B}}_{u,l}^{o}{\mathbf {B}}_{u,l}^{o,H}{\mathbf {H}}_{u,l}^{H}\mathbf {T}_{l}^{H}{} \mathbf{{S}}_{u}^{H}\right| \\ {}&=\sum _{l=1}^{U_u}\log _{2}\left| \mathbf {I}_{N_{u,l}}+{\frac{{1}}{K+1}}\times \mathbf{{S}}_{u}\mathbf {T}_{l}{\mathbf {D}}_{u}{\mathbf {B}}_{u,l}^{o}{\mathbf {B}}_{u,l}^{o,H}{\mathbf {D}}_{u}^{H}\mathbf {T}_{l}^{H}{} \mathbf{{S}}_{u}^{H}\right| \end{aligned} \end{aligned}$$

where $$\left( \sigma _{\tilde{v}}^2\mathbf {I}_{N_{u,l}} +\sum _{i\ne {l}}^{U_{u}}\mathbf {T}_{l}{\mathbf {H}}_{u,i}{\mathbf {B}}_{u,i}^{o}{\mathbf {B}}_{u,i}^{o,H}{\mathbf {H}}_{u,i}^{H}\mathbf {T}_{l}^{H}\right) ^{-1}=\mathbf{{S}}_{u}^{H}{} \mathbf{{S}}_{u}$$ and $${\mathbf {D}}_{u}=\sqrt{K+1}{\mathbf {H}}_{u,l}=\sqrt{K}{\bar{\mathbf {H}}_{u,l}}+{\tilde{\mathbf {H}}_{u,l}}\sim ~\mathscr{CN}\mathscr{}\left( \sqrt{K}\bar{\mathbf {H}}_{u,l},{\sigma _{v}^2}{\mathbf {I}}_{{N}_{r}}\right)$$.

The relationship between the Rician factor *K*, and the multipath link and channel capacity found in result 1 is maintained in result 2 as well.

At last, the total spectral efficiency of the FD Multiuser MIMO mode of communication is expressed by making use of (22) and (39) as shown as

40 $$\begin{aligned} {\mathrm {R}}_{T}={{\mathrm R}}_{d}+{\mathrm {R}}_{u}. \end{aligned}$$

The total SE of the half-duplex operating BS is given by half of the aggregate of the uplink achievable sum rate without the effect of self-interference and downlink sum rate.

## Energy efficiency maximization

A communication system’s Energy Efficiency transmission design depends on circuit power, as well as the actual transmitted power allocated for data transmission, radiated by electronic devices. A power consumption model is provided to address the optimization issue of FD-MU-MIMO system EE optimization.

### Power consumption model

There are several hardware elements involved in data communication that consume energy when transmitting data. Thus, accurate modeling of total power consumption plays a crucial role in energy-efficient designs^41,42^. As shown in^43,44^, the power dissipation consists of the transmission and the circuit power. In our system model, both uplink and downlink power consumption are accounted for. With this approach, it is possible to determine the total power used by the base station for the DL channel

41 $$\begin{aligned} \mathrm {P}_{d,T}=\frac{1}{\xi }{\mathrm {P}_{tr}}+\mathrm {{P}_{d}^{C}}=\frac{1}{\xi }\sum _{l=1}^{U_d}{\mathrm Tr}\left( {\mathbf {A}_{d,l}}{\mathbf {A}}_{d,l}^{H}\right) +{N_{t}}{\mathrm {P}_{d}^{\mathrm {dyn}}}+\mathrm {P}_{d}^{\mathrm {sta}} \end{aligned}$$

in which $\xi {\in }~(0, 1]$ refers to the power amplifier’s efficiency. ${\mathrm {P}_{tr}}$ is the transmit power obtained by linear precoders, $\mathrm {{P}_{d}^{C}}={N_t}{\mathrm {P}_{d}^{\mathrm {dyn}}}+\mathrm {P}_{d}^{\mathrm {sta}}$ stands for circuit power. Additionally, ${\mathrm {P}_{d}^{\mathrm {dyn}}}$ represents the dynamic circuit power consumption related to the power radiation of all circuits which scales linearly with the number of the transmit antennas, and $\mathrm {P}_{d}^{\mathrm {sta}}$ is the static circuit power.

Similar to this, the total power consumed by the $r\mathrm{th}$ user’s transmitter in the UL channel is denoted as

42 $$\begin{aligned} \mathrm {P}_{u,T}=\frac{1}{\xi }{\mathrm {P}_{u}}+\mathrm {{P}_{u}^{C}}=\frac{1}{\xi }{\mathrm Tr}\left( {\mathbf {B}_{u,l}}{\mathbf {B}}_{u,l}^{H}\right) +{N_{r}}{\mathrm {P}_{u}^{\mathrm {dyn}}}+\mathrm {P}_{u}^{\mathrm {sta}} \end{aligned}$$

where $\xi {\in }~(0, 1]$ denotes the power amplifier’s efficiency and ${\mathrm {P}_{u}}$ denotes the transmit power designated for data stream transmission. As well, $\mathrm {{P}_{u}^{C}}$ refers to the circuit power and represented as $N_r{\mathrm {P}_{u}^{\mathrm {dyn}}}$ plus $\mathrm {P}_{u}^{\mathrm {sta}}$, in which ${\mathrm {P}_{u}^{\mathrm {dyn}}}$ represents the dynamic circuit power and $\mathrm {P}_{u}^{\mathrm {sta}}$ denotes the static circuit power.

### Problem formulation and proposed EE maximization

By dividing both DL and UL channels’ sum rates by the overall system power consumption, we find the EE metric, which is measured in bit/Hz/joule. Based on this definition, the overall achievable EE of the FD-MU-MIMO system considered can be expressed as follows

43 $$\begin{aligned} \begin{aligned} \mathrm {EE}&=\frac{\mathrm {SE}}{\mathrm {Total~power~consumption}}=\frac{R_{d} +{R}_{u}}{{\frac{1}{\xi }\sum _{l=1}^{U_d}{\mathrm Tr}\left( {\mathbf {A}_{d,l}}{\mathbf {A}}_{d,l}^{H}\right) +\frac{1}{\xi }\sum _{l=1}^{U_u}{\mathrm Tr}\left( {\mathbf {B}_{u,l}}{\mathbf {B}}_{u,l}^{H}\right) +\mathrm {{P}^{C,T}}}} \end{aligned} \end{aligned}$$

in which $\mathrm {{P}^{C,T}}=\mathrm {{P}_{d}^{C}}+\mathrm {{P}_{u}^{C}}$ stands for the total circuit power of the system. For analytical purposes, the circuit power $\mathrm {{P}^{C,T}}$ is treated as a constant and is summarized in^45,46^. The following EE design optimization problem is thus addressed

44 $$\begin{aligned} \begin{aligned}{}&\max \limits _{{\mathbf {A}}_{d,l},{{\mathbf {B}}_{u,l}}}{{\mathrm EE}}\\ {}&{{\mathrm s.t.}} \quad \sum _{l=1}^{U_d} {\mathrm Tr}\left( {\mathbf {A}}_{d,l}{\mathbf {A}}_{d,l}^{H}\right) \le {P_{tr}}\\ {}&\quad \quad {\mathrm Tr}\left( {\mathbf {B}}_{u,l}{\mathbf {B}}_{u,l}^{H}\right) \le {P_{u,l}},\quad l={1,2,\ldots ,{U_u}}. \end{aligned} \end{aligned}$$

Now, we recall (21) and (39) then re-write (43) as

45 $$\begin{aligned} \begin{aligned} \mathrm {EE}&=\frac{1}{P_R}\bigg (\left( \sum _{l=1}^{U_d}\log _{2}\left| \mathbf {I}_{N_{d,l}}+{\mathbf {A}}_{d,l}^{o,H}{\mathbf {H}}_{d,l}^{H}\mathbf {Q}_{l}^{H}{} \mathbf{{S}}_{d}^{H}{} \mathbf{{S}}_{d}\mathbf {Q}_{l}{\mathbf {H}}_{d,l}{\mathbf {A}}_{d,l}^{o}\right| \right) +\\ {}&\left( \sum _{l=1}^{U_u}\log _{2}\left| \mathbf {I}_{N_{u,l}}+{\mathbf {B}}_{u,l}^{o,H}{\mathbf {H}}_{u,l}^{H}\mathbf {T}_{l}^{H}{} \mathbf{{S}}_{u}^{H}{} \mathbf{{S}}_{u}\mathbf {T}_{l}{\mathbf {H}}_{u,l}{\mathbf {B}}_{u,l}^{o}\right| \right) \bigg )\\ {}&=\frac{1}{P_R}\left( \left( \sum _{l=1}^{U_d}\log _{2}\left| \mathbf {I}_{N_{d,l}}+\mathbf{{C}}_{d}^{H}\widetilde{\mathbf {Q}}_{l}^{-1}{} \mathbf{{C}}_{d}\right| \right) + \left( \sum _{l=1}^{U_u}\log _{2}\left| \mathbf {I}_{N_{u,l}}+\mathbf {W'}^{H}\widetilde{\mathbf {V}}_{l}^{-1}\mathbf {W'}\right| \right) \right) \end{aligned} \end{aligned}$$

where ${P_R}={{\frac{1}{\xi }\sum _{l=1}^{U_d}{\mathrm Tr}\left( {\mathbf {A}_{d,l}}{\mathbf {A}}_{d,r}^{H}\right) +\frac{1}{\xi }\sum _{l=1}^{U_u}{\mathrm Tr}\left( {\mathbf {B}_{u,l}}{\mathbf {B}}_{u,l}^{H}\right) }}+\mathrm {{P}^{C,T}}$. Due to the fact that $\widetilde{\mathbf {Q}}_{l}^{-1}$ and $\widetilde{\mathbf {V}}_{l}^{-1}$ are positive definite, $\left( \sigma _{\tilde{v}}^{2}\mathbf {I}_{N_{d,l}} +\sum _{i\ne {l}}^{U_{d}}{\mathbf {Q}_{l}}{\mathbf {H}}_{d,l}{\mathbf {A}}_{d,i}^{o}{\mathbf {A}}_{d,i}^{o,H}{\mathbf {H}}_{d,l}^{H}\mathbf {Q}_{l}^{H}\right) ^{-1}=\widetilde{\mathbf {Q}}_{l}^{-1}=\mathbf{{S}}_{d}^{H}{} \mathbf{{S}}_{d}$, $\left( \sigma _{\tilde{v}}^2\mathbf {I}_{N_{u,l}} +\sum _{i\ne {l}}^{U_{u}}\mathbf {T}_{l}{\mathbf {H}}_{u,i}{\mathbf {B}}_{u,i}^{o}{\mathbf {B}}_{u,i}^{o,H}{\mathbf {H}}_{u,i}^{H}\mathbf {T}_{l}^{H}\right) ^{-1}=\widetilde{\mathbf {V}}_{l}^{-1}=\mathbf{{S}}_{u}^{H}{} \mathbf{{S}}_{u}$, $\mathbf{{C}}_{d}^{H}={\mathbf {A}}_{d,l}^{o,H}{\mathbf {H}}_{d,l}^{H}\mathbf {Q}_{l}^{H}$ and $\mathbf {W'}^{H}={\mathbf {B}}_{u,l}^{o,H}{\mathbf {H}}_{u,l}^{H}\mathbf {T}_{l}^{H}$. As well, $\mathbf{{C}}_{d}^{H}{} \mathbf{{S}}_{d}^{H}{} \mathbf{{S}}_{d}{} \mathbf{{C}}_{d}=\mathbf{{C}}_{d}^{H}\widetilde{\mathbf {Q}}_{l}^{-1}{} \mathbf{{C}}_{d}=\mathbf {U'}\Delta \mathbf {U'}^{H}$ where $\Delta =\mathrm {diag}\left( \iota _{l,1},\ldots ,\iota _{l,N_{d,l}}\right)$ in which each diagonal entry becomes the eigenvalue of $\mathbf{{C}}_{d}^{H}\widetilde{\mathbf {Q}}_{l}^{-1}{} \mathbf{{C}}_{d}$ and $\mathbf {U'}=\left[ \mathbf {u'_1}~\mathbf {u'_2}~\cdots ~\mathbf {u'}_{N_{d,l}}\right]$ represents a Hermitian matrix where each column is the corresponding eigenvector. In addition, $\mathbf {W'}^{H}{} \mathbf{{S}}_{u}^{H}{} \mathbf{{S}}_{u}\mathbf {W'}=\mathbf {W'}^{H}\widetilde{\mathbf {V}}_{l}^{-1}\mathbf {W'}={\mathbf {U''}}\Omega \mathbf {U''}^{H}$ where $\Omega =\mathrm {diag}\left( \upsilon _{r,1},\ldots ,\upsilon _{l,N_{r}}\right)$ each diagonal element is the eigenvalue of $\mathbf {W'}^{H}\widetilde{\mathbf {V}}_{l}^{-1}\mathbf {W'}$ and $\mathbf {U''}=\left[ \mathbf {u''_1}~\mathbf {u''_2}~\cdots ~\mathbf {u''}_{N_{r}}\right]$ is a Hermitian matrix where each column is the corresponding eigenvector. As a result, when all of the eigenvalues of $\mathbf{{C}}_{d}^{H}\widetilde{\mathbf {Q}}_{l}^{-1}{} \mathbf{{C}}_{d}$ and $\mathbf {W'}^{H}\widetilde{\mathbf {V}}_{l}^{-1}\mathbf {W'}$ much greater than 1, we have $\left| \mathbf {I}_{N_{d,l}}+\mathbf{{C}}_{d}^{H}\widetilde{\mathbf {Q}}_{l}^{-1}{} \mathbf{{C}}_{d}\right| \approx \left| \mathbf{{C}}_{d}^{H}\widetilde{\mathbf {Q}}_{l}^{-1}{} \mathbf{{C}}_{d}\right| =\left| \mathbf{{C}}_{d}{} \mathbf{{C}}_{d}^{H}\widetilde{\mathbf {Q}}_{l}^{-1}\right|$ as well as $\left| \mathbf {I}_{N_{u,l}}+\mathbf {W'}^{H}\widetilde{\mathbf {V}}_{l}^{-1}\mathbf {W'}\right| \approx \left| \mathbf {W'}^{H}\widetilde{\mathbf {V}}_{l}^{-1}\mathbf {W'}\right| =\left| \mathbf {W'}\mathbf {W'}^{H}\widetilde{\mathbf {V}}_{l}^{-1}\right|$. Thus, (45) can be rewritten as

46 $$\begin{aligned} \begin{aligned} \mathrm {EE}&\approx \sum _{l=1}^{U_d}\log _{2}\left[ \left| \mathbf{{C}}_{d}{} \mathbf{{C}}_{d}^{H}\widetilde{\mathbf {Q}}_{l}^{-1}\right| ^{\frac{1}{P_R}}\right] +\sum _{l=1}^{U_u}\log _{2}\left[ \left| \mathbf {W'}\mathbf {W'}^{H}\widetilde{\mathbf {V}}_{l}^{-1}\right| ^{\frac{1}{P_R}}\right] \bigg ). \end{aligned} \end{aligned}$$

## Simulation results and analysis

This section provides simulation results for Half-duplex Multiuser MIMO and Full-duplex Multiuser MIMO communications by SLNR beamforming technique for small cell deployments in^47–49^. The channels ${\mathbf {H}}_{d,l}$ and ${\mathbf {H}}_{u,l}$ are generated as Rician fading distributions. Additionally, the SI channel is distributed as ${\mathbf {H}}_{SI,l}\sim ~\mathscr{CN}\mathscr{}\left( \sqrt{\frac{{K_1{\sigma _{SI}^2}}}{K_1+1}}{\bar{\mathbf {H}}_{SI,l}},{\frac{{\sigma _{SI}^2}}{K_1+1}}{\mathbf {I}}_{{N}_{r}{{N}_{t}}}\right)$ with a small Rician factor to characterize the residual SI channel after SI cancellation techniques according to^47,50,51^. $\sigma _{SI}^2$ is added to parameterize the potentiality of particular self-interference cancellation mechanisms, $K_1=1$ is the Rician factor without loss of generality and ${\bar{\mathbf {H}}_{SI,l}}$ is the LOS matrix with a magnitude of all ones. This paper assumed the maximum transmitted power at every user to be equal for all users that is $P_{ul,l}=P_{ul}$ and the noise power is given by $\sigma _{v,l}^2={\sigma _{\tilde{v}}^2}=1$ for $l=1,2,\ldots ,U$, where $U=U_u$ is for the UL channel and $U=U_d$ is for the DL channel. We assumed the number of users $U_d=U_u=2$ and the number of streams per user $m = n=2$ for both channels. The total number of antennas at the half-duplex system is taken $N_{TR}=N_t+N_r$. The results are acquired by averaging 10,000 channel realizations. As well, Table 1 shows the default parameters for the simulations.

**Table 1:** Default simulation parameters.

Parameters	Values	Parameters	Values
LOS matrix, $\bar{\mathbf {H}}_{d,l}=\bar{\mathbf {H}}_{u,l}$	Entries of magnitude of all ones	Users, $U_d=U_u$	2
Streams per user, $m = n$	2	Max power at BS $(P_{tr})$	10 dBm, 26 dBm
Noise power, ${\sigma _{\tilde{v}}^2}=\sigma _{v}^2$	1	Max power per user $(P_{ul}=P_{ul,l})$	10 dBm, 23 dBm

**Figure 2:**
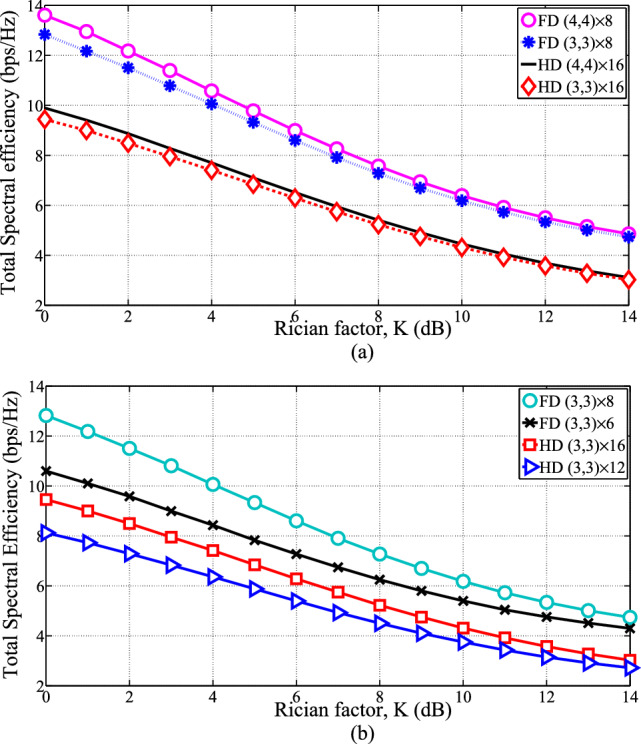
Total SE versus Rician factor for Full-duplex and Half-duplex systems for: (**a**) varying number of antennas at users $N_{d,l}=N_{u,l}=3$ and 4, $N_t=N_r=8$ and $N_{TR}=16$. (**b**) $N_{d,l}=N_{u,l}=3$ and varying number of antennas at the base station $N_t=N_r=6$ and 8.

Figure 2 shows the total SE comparisons of FD Multiuser MIMO and HD Multiuser MIMO mode of communications for two cases by setting $\sigma _{SI}^2=-\,80$ dB. Case 1: we vary the number of antennas per user from $N_{d,l}=N_{u,l}=3$ to $N_{d,l}=N_{u,l}=4$ and set the number of antennas at the BS $N_{t}=N_{r}=8$ as shown in Fig. 2a. Case 2: we increase the number of antennas at the base station from $N_{t}=N_{r}=6$ to $N_{t}=N_{r}=8$ and fix the number of antennas at every user $N_{d,l}=N_{u,l}=3$ as illustrated in Fig. 2b. Increasing the number of antennas at the BS and both channel users, as illustrated in Fig. 2, results in an improvement in the SE of both FD and HD systems because more degrees of freedom in the spatial domain can be used to strengthen the signal power received by the target receiver. For both cases, the figure validates the significant enhancement in SE of FD over HD for all *K* (Rician factor) and for the power transmitted constraints at the base-station $P_{tr}=26$ dBm plus at uplink channel users $P_{ul}=23$ dBm based on studies^48,49^. This is because of the precoding scheme called SLNR for Full-duplex Multi-user MIMO communications can utilize all channel degrees of freedom. Nevertheless, the total spectral efficiencies of HD and FD systems drastically decrease as the Rician factor increase and for a fixed transmitted power. This is because the Rician fading resembles an AWGN channel. Additionally, a strong line-of-sight link reduces the channel capacity, i.e., MIMO communications merit from richly scattering surroundings. Even though the SE of the FD-MU-MIMO mode system decreases as the Rician factor grows, even now this offers good performance as compared to the traditional Half-duplex MU-MIMO ways of the communication system.

**Figure 3:**
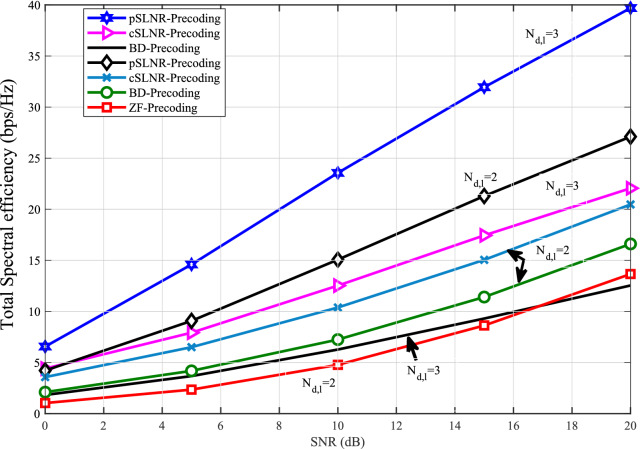
Total SE versus SNR for the downlink FD MU-MIMO communications for the precoding schemes BD, ZF, cSLNR and pSLNR for $P_{ul}$ = 23 dBm, Rician factor $K=1$, $N_{d,l}=2~\text {and}~ 3$ per user, and $N_t=4$.

Figure 3 illustrates the SE comparisons of the proposed SLNR (pSLNR) precoding scheme, conventional SLNR (cSLNR), ZF, and BD precoding methods on the Rician fading surrounding. It is important to note that the computational complexity of the leakage-based solution and the ZF approach is similar, i.e., $O(N_{t}^3)$ as shown in^28^. For all given SNR values, the SE of downlink Full-duplex Multi-user MIMO systems according to the proposed technique performs well relative to the precoding schemes mentioned. It is also shown that the spectral efficiencies of both conventional SLNR and proposed SLNR precoding schemes rise for the number of antennas for each user increases from $N_{d,l} = 2$ to $N_{d,l} = 3$ and for a given number of antennas at the BS $N_{t} = 4$. However, for the case of BD precoding, the SE is worse for the number of antennas for each user is $N_{d,l} = 3$ and the number of antennas at the BS is $N_{t} = 4$. This capacity loss is observable due to dimensional restrictions such that every user can only transmit one data stream. In addition, this indicates that it cannot take advantage of all the channel d.o.f (degrees of freedom). Furthermore, for the case of ZF precoding, the SE fails for $N_{d,l} = 3$ and $N_{t} = 4$. This is because the ZF scheme should not fulfill the requirement that the number of transmitter antennas at the base station becomes approximately higher than the sum of all receiver antennas at users. As a result, the proposed and conventional precoding schemes based on SLNR techniques attain high spectral efficiency gains. As well, they overwhelm the dimensional restrictions on Block-diagonalization and Zero-forcing precoding schemes.

**Figure 4:**
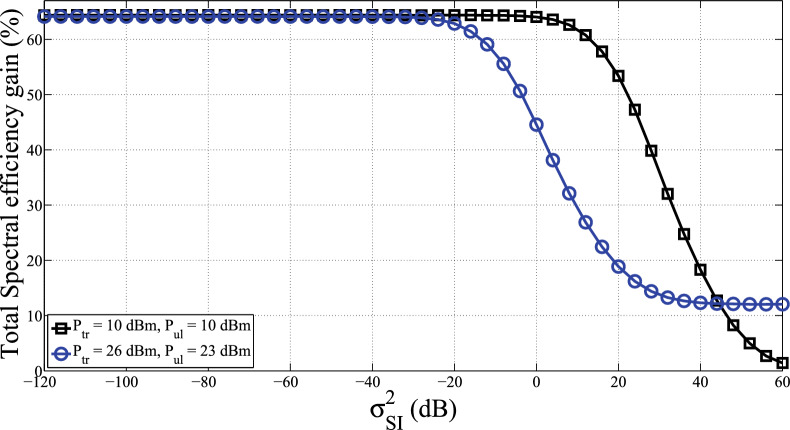
Total spectral efficiency percentage gain versus $\sigma _{SI}^2$, for $N_{TR}=16$, $N_t=N_r=8$, $N_{d,l}=N_{u,l}=4$  and  *K*-factor $=1$.

Figure 4 depicts the total spectral efficiencies percentage gains of Half-duplex over Full-duplex communications by varying the power transmitted for two scenarios under 3GPP LTE for small cell deployments according to^47,49,50^. The first scenario is for the transmitted power at the uplink users is $P_{ul}=23$ dBm again $P_{tr}=26$ dBm at the base station. The second scenario is for the transmitted power at the BS and uplink users are the same, i.e., $P_{tr} = P_{ul}=10$ dBm. The SE gains of FD compared to HD for both scenarios are around 64.4% as $\sigma _{SI}^2\le 5$ dB for $P_{tr} =P_{ul}=10$ dBm and as $\sigma _{SI}^2\le -25$ for $P_{tr}=26$ dBm and $P_{ul}= 23$ dBm. However, the SE gains are drastically decreasing when $\sigma _{SI}^2>-20$ dB for the first scenario and when $\sigma _{SI}^2>5$ dB for the second scenario. This is because high power transmitted at the BS generates substantial self-interference signal power as well eminent transmitted power at the uplink users produces powerful CCI power. This indicates that reducing transmit power of users in the uplink channel results in decreasing the CCI. Accordingly, the sum rate of the downlink transmission has increased. Moreover, reducing the transmit power at the BS results in reduced self-interference. Therefore, the sum rate in the uplink transmission increased. The figure shows that the SE gain of a full-duplex system is higher when the transmit powers are smaller. It is because smaller transmit powers produce less self-interference and CCI. Furthermore, Fig. 4 reveals when the self-interference is infirm, the gain becomes saturated by the noise. The reason for this is that the signal-to-leakage-and-noise ratio scheme is allied to the conventional beamforming technique which, does not take into account self-interference.

**Figure 5:**
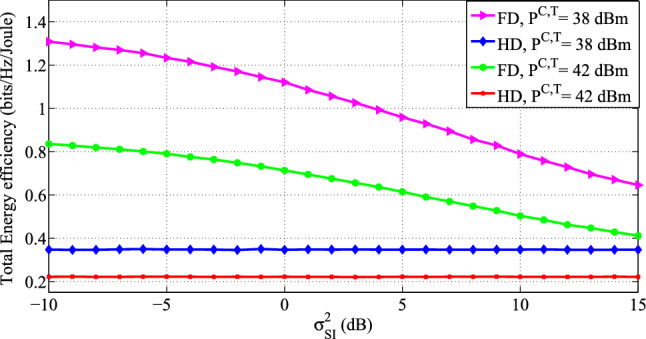
Total energy efficiency versus $\left( \sigma _{SI}^2 \right)$ for $N_{TR}=16$, $N_{t}=N_{r}=8$ and $N_{d,l}=N_{u,l}=4$.

The attainable EE for FD MU MIMO and HD-MU MIMO systems with different amounts of transmit antennas at the BS and users for specific values of $\mathrm {{P}^{C,T}}$ is shown in Fig. 5. We set $P_{u}= 23$ dBm, Ricean-factor $K=1$ and $P_{tr}=26$ dBm. The plot clearly shows that the energy efficiency of both systems decreases as circuit power consumption increases. This is because increased $\mathrm {{P}^{C,T}}$ results in higher energy consumption. The results indicated that, even in the presence of significant SI, the FD system provided EE improvements over the HD system. This is because of the proper design of the precoding transmission scheme for the FD-assisted system.

## Conclusion

The SE of FD related to the HD mode of communications on the Rician fading environment has been effectively optimized in this research using an improvement to the signal-to-leakage-and-noise ratio (SLNR)-based precoder design. Our research demonstrates enhancing the SLNR downlink transmission technique with CCI awareness. To reduce interferences, this method uses a whitening filter at the receiver. Yet again, we design a precoder using CCI plus noise covariance matrices. We also propose an SI-aware advancement based on the SLNR technique as well as design a new precoder using the self-interference plus noise covariance matrices for the uplink channel. Additionally, this system employs a suppression filter for interference mitigation. The precoder design based on the SLNR criterion decouples multi-streams and gives a closed-form solution. Further, the proposed SLNR precoding scheme supports enormous users plus holds up many antennas at the BS and both channel users simultaneously compared to zero-forcing and block diagonalization precoding schemes. Therefore, the FD-based way of communication systems using the SLNR precoding technique is an encouraging technique for future generation small cell systems. Furthermore, we use a power consumption model to achieve maximum energy efficiency. Numerical results validate the SE of Full-duplex Multiuser MIMO is eminently improved compared with Half-duplex MU-MIMO when the number of antennas of each user raises for all Rician factors and a fixed number of antennas at the base station. Moreover, the spectral efficiency gain of the FD mode of transmission is better than HD when the maximum transmitted power at users and BS are small. The reason for that is smaller transmitting powers generate smaller signal powers of the SI and CCI. We also show that the attainable EE of FD is higher than the existing HD system by using the proposed SLNR approach for different values of transmit power and circuit power consumption.

## Data Availability

All data generated or analyzed during this study are included in this submitted article.
